# The impact of salt-tolerant plants on soil nutrients and microbial communities in soda saline-alkali lands of the Songnen plain

**DOI:** 10.3389/fmicb.2025.1592834

**Published:** 2025-06-05

**Authors:** Junjie Song, Xueting Guan, Haojun Cui, Lin Liu, Yan Li, Yuhua Li, Shurong Ma

**Affiliations:** Key Laboratory of Saline-Alkali Vegetation Ecology Restoration, Ministry of Education, College of Life Science, Northeast Forestry University, Harbin, China

**Keywords:** salt tolerant plants, soda saline-alkali soil, soil physical and chemical properties, rhizosphere microorganisms, driving factors

## Abstract

**Introduction:**

Soil salinization poses a significant threat to agricultural development and ecosystem health. While phytoremediation is an effective approach, the mechanisms by which salt-tolerant plants mediate saline-alkali soil amelioration in the Songnen Plain remain unclear.

**Methods:**

We selected seven common salt-tolerant plants from the Songnen Plain to compare soil nutrients and microbial communities between vegetated areas and bare alkali patches. Soil salinity, pH, nutrient content (TN, TP, TK), enzyme activities (Catalase, Cellulase, Saccharase, Urease), and microbial structure were analyzed.

**Results:**

Plant presence significantly reduced soil salinity and pH while increasing nutrient levels and enzyme activities, with SL (*Salix linearistipularis*) showing the most comprehensive improvement. Vegetation enhanced microbial abundance/diversity (bacteria and fungi), with EC (R^2^ = 0.7308, *P* = 0.0001) and TN (R^2^ = 0.5706, *P* = 0.0001) as key drivers of diversity changes. Microbial composition shifted markedly: beneficial phyla (e.g., Mortierellomycota, Acidobacteriota) increased, while pathogenic Chytridiomycota decreased. Community variations were primarily influenced by EC (R^2^ = 0.8778, *P* = 0.001) and pH (R^2^ = 0.8661, *P* = 0.001). Plants also promoted functional groups involved in carbon/nitrogen cycling.

**Discussion:**

These findings demonstrate that salt-tolerant plants enhance soil fertility and restructure microbial communities in saline-alkali soils, with EC/pH and TN as critical regulatory factors. This study provides a scientific basis for sustainable saline land rehabilitation via targeted vegetation restoration.

## 1 Introduction

Soil salinization has become one of the most significant global environmental and economic issues, severely threatening food security and ecosystem health. It adversely affects plant growth, crop yield, soil structure, and fertility (Hassani et al., 2021; Tarolli et al., 2024; Zhang et al., 2024a). In China, the area of saline-alkali land reaches 99 million hectares (Chen et al., 2024). The Songnen Plain in Northeast China, with over 3 million hectares affected by salinization, is one of the world's three major concentrated distribution areas of soda saline-alkali land (Yang et al., 2016). The salts in soda saline-alkali land are primarily composed of NaHCO3 and Na_2_CO3, characterized by high pH and rich soil colloid content. This results in simultaneous salinization and alkalization, making the soil in this region more barren and unsuitable for cultivation, severely restricting agricultural development (Chi et al., 2011; Li et al., 2016). Against the backdrop of the global food crisis, how to effectively utilize saline-alkali land and transform it into fertile farmland has become a hot research topic. Current methods for improving saline-alkali land mainly include physical approaches (irrigation and drainage, soil replacement), chemical methods (application of gypsum, humic acid), and biological methods (phytoremediation, microbial remediation) (Elmeknassi et al., 2024; Heng et al., 2022; Hu et al., 2024). Physical methods require significant labor, while chemical methods may cause secondary pollution (Wang et al., 2024a; Zhang et al., 2024b). In contrast, biological remediation not only offers a low-cost solution for soil improvement but also reduces the risk of soil pollution, making it a key direction in current research on saline-alkali soil remediation (Hu et al., 2024; Jiang et al., 2015).

Halophytes are naturally salt-tolerant plants that can endure salinity levels intolerable to most plants, thriving and surviving in saline soils. Halophytes and salt-tolerant plants have evolved various adaptive mechanisms to cope with salt-alkali stress, including salt gland secretion, regulation of cellular ion homeostasis and osmotic pressure, detoxification of reactive oxygen species, and alterations in membrane composition (Flowers and Colmer, 2008; Meng et al., 2018). With the continuous expansion of saline-alkali land, the sustainable development of traditional agriculture faces significant challenges. Phytoremediation is a sustainable and cost-effective approach. Cotton/halophytes can reduce soil salinity through intercropping, improving soil physicochemical properties and crop yield, thereby generating higher economic benefits (Liang and Shi, 2021). Salt-tolerant legumes such as *Glycine soja* and *Sesbania cannabina* have successfully rehabilitated saline soils by reducing soil electrical conductivity (EC) and accumulating carbon and nitrogen, enriching microbial communities at different soil depths (Zheng et al., 2023). Furthermore, plant cultivation not only ameliorates saline-alkali soils but also enhances ecosystem multifunctionality (EMF), increasing microbial community richness and network complexity (Hu et al., 2024; Qiu et al., 2022).

Soil microorganisms are crucial for the sustainable development of ecosystems (Zhang et al., 2024a). They facilitate the decomposition of plant litter, the formation of humus, and play a significant role in biogeochemical cycles (Djukic et al., 2010). In natural environments, soil microorganisms are highly sensitive to environmental changes and are easily influenced by factors such as soil salinity, nutrients, and pH. Therefore, the structure and diversity of soil microbial communities serve as important indicators of soil health and ecosystem stability (Fierer et al., 2021; Liu et al., 2020; Yang et al., 2024). Soil electrical conductivity (EC) and salt ion content are considered primary factors affecting soil microorganisms. Increases in soil EC and salinity significantly reduce microbial diversity and richness, altering microbial community structure (Guan et al., 2021; Guo et al., 2023; Zhang et al., 2024a, 2021). Compared to bulk soil, the plant rhizosphere actively shapes a distinct microenvironment through continuous secretion of organic compounds (e.g., sugars, organic acids) (Kumar et al., 2023), characterized by: enhanced nutrient availability; and generally reduced pH and EC values (Balyan and Pandey, 2024; Ström et al., 2002). This plant-mediated niche construction drives significant microbial community shifts, manifesting as: increased abundance of exudate-utilizing taxa (Vives-Peris et al., 2020); and elevated overall microbial diversity (Qiu et al., 2022), though with potential reduction of halophilic specialists (Wang et al., 2020). Concurrently, rhizosphere microbes engage in bidirectional interactions with plants through signal exchange, ultimately enhancing plant stress tolerance (Gupta et al., 2021; Peng et al., 2023).

The root system of plants serves as the primary source of nutrient acquisition and represents the most dynamic microenvironment in the soil. In this region, plant-microbe-environment interactions collectively maintain ecosystem stability. However, the mechanisms underlying the interactions between microbial communities and plants remain unclear, hindering our understanding of how salt-tolerant plants influence microbial community assembly. The Songnen Plain, one of the world's three major soda saline-alkali regions, exhibits severe salinization but supports a variety of salt-tolerant plants. Nevertheless, the impact of these plants on the microbial communities in saline-alkali soils is not well understood. Therefore, in this study, we evaluated the remediation effects of these plants on saline-alkali soils by measuring the physicochemical properties and enzyme activities of rhizosphere soils and alkali patches under different salt-tolerant plants. Using high-throughput sequencing, we investigated the effects of salt-tolerant plants on the richness and diversity of microbial communities. Finally, we identified key environmental factors influencing microbial community diversity and composition, as well as the functional group dynamics of microbial communities.

## 2 Materials and methods

### 2.1 Study site and soil sampling

**Figure 1:**
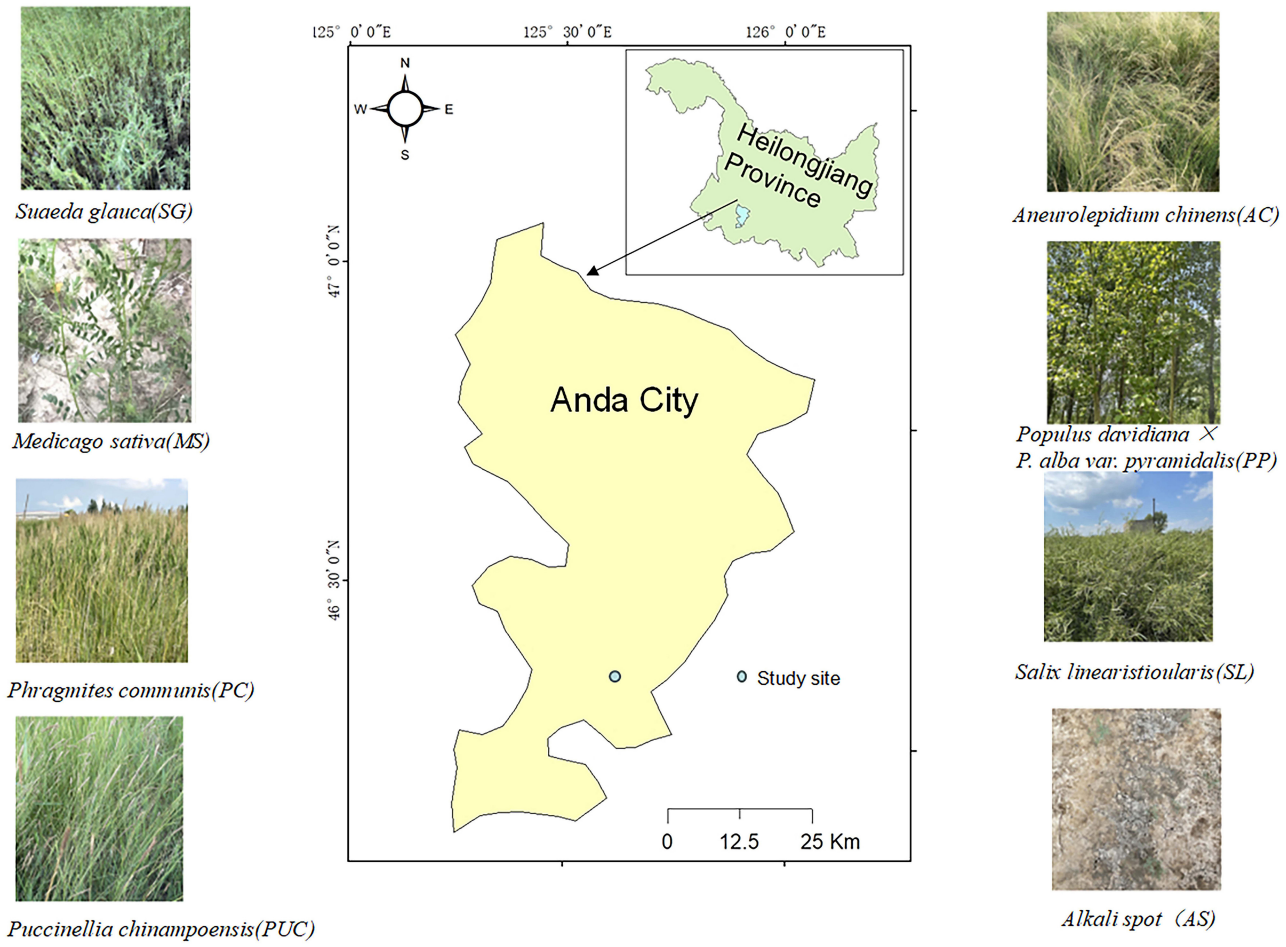
Schematic diagram of sampling area and vegetation at sampling sites.

The study area is located in Anda City, Heilongjiang Province (46°4′-47°1′N, 124°53′-125°55′E). Sampling was conducted within the experimental station of Northeast Forestry University in Anda (Figure 1). Established in 2002, the station initiated large-scale phytoremediation of saline-alkali soils in 2005, with continuous restoration efforts until the sampling year (2020), totaling 15 years of intervention. The sampling area covered approximately 6 hectares, characterized by flat terrain (slope < 2°) and uniform sandy loam saline-alkali soil, with consistent historical management practices (no fertilization or irrigation). The region experiences a northern temperate continental semi-arid monsoon climate, with a mean annual temperature of 4.2°C, a frost-free period of 129 days, and an average annual precipitation of 432.5 mm, of which approximately half occurs during summer. Prior to restoration, the area exhibited severe saline-alkalization with relatively simple vegetation community structure.

For sampling design, quadrats (1 m × 1 m) were randomly established for seven plant species—*Phragmites communis* (PC), *Puccinellia chinampoensis* (PUC), *Medicago sativa* (MS), *Aneurolepidium chinense* (AC), *Suaeda glauca* (SG), *Salix linearistipularis* (SL), and *Populus davidiana* × *P. alba var. Pyramidalis* (PP), with Alkaline spot (AS, bare soil patches with pH >10 and EC >5.3 dS/m) as the control. To minimize interspecific interference and local soil heterogeneity, a minimum spacing of ≥15 meters was maintained between quadrats. For each plant species, three replicate quadrats were established, with rhizosphere soil samples (0–20 cm depth) collected using a soil auger at five points (four corners and center) per quadrat. After removing roots and debris, samples from each quadrat were homogenized to form a composite sample, from which 1 kg subsamples were obtained by quartering and stored in sterile ziplock bags. During sampling, microtopographic consistency (no significant elevation differences) was ensured across all plant quadrats and control spots, with surrounding vegetation cover (absence of tree interference) documented. All soil samples were immediately stored in iceboxes and transported to the laboratory within 24 h. Each sample was divided into two aliquots: one preserved at −80°C for high-throughput sequencing, and the other air-dried in a ventilated area, then ground and sieved through a 2-mm mesh (with roots and stones removed) for physicochemical property and enzyme activity analyses.

### 2.2 Determination of soil physicochemical properties and enzyme activities

Soil pH was measured using a pH meter (FE28, METTLER-TOLEDO, USA) at a soil-to-water ratio of 1:2.5 (m/v). Soil electrical conductivity (EC) was determined using a conductivity meter (Bante902P, Bante, CN) at a soil-to-water ratio of 1:5 (m/v). Soil total nitrogen (TN) was assayed using the Kjeldahl method. Soil total potassium (TK) was quantified via an ammonium acetate extraction flame photometric method (JHR-2, Ye Tuo, Shanghai). Total phosphorus (TP) was determined by the colorimetric method (JHR-2, Ye Tuo, Shanghai). Soil organic matter (SOM) was measured using the spectrophotometric method. Soil ${\text{NO}}_{3^{-}}$-N and ${\text{NH}}_{4^{+}}$-N were determined by the micro method. Soil bulk density (BD) and soil water content (SWC) were measured using the ring knife method and the drying-weighing method, respectively (Chen et al., 2021b; Kang et al., 2002). The activities of soil urease, alkaline phosphatase, catalase, and sucrase were measured using kits provided by Nanjing Convinced Testing Technology Corporation (Nanjing, China). All tests were conducted in triplicate, and the final results were averaged.

### 2.3 DNA extraction, amplicon sequencing and data processing

Bacterial 16S rRNA sequences were amplified using forward primer 338F (5′-ACTCCTACGGAGGCAGCAG-3′) and reverse primer 806R (5′-GGACTACHVGGGTWTCTAAT-3′; Caporaso et al., 2011). Fungal ITS sequences were amplified with forward primer ITS1F (5′-CTTGTCATTTAGAGGAAGTAAGTAA-3′) and reverse primer ITS2R (5′-GCTGCGTTCTTCATCGATGC-3′; Chen et al., 2021a). PCR was performed in 20 μl reactions containing 10 μl 2 × Pro Taq mix, 0.8 μl of each primer (5 μM), and 10 ng template DNA, with ddH_2_O added to a final volume of 20 μl. The amplification protocol consisted of initial denaturation at 95°C for 5 min, followed by 30 cycles of denaturation at 95°C for 30 s, annealing at 55°C for 30 s, and extension at 72°C for 45 s, with a final extension at 72°C for 5 min. PCR products were verified by 1.5% agarose gel electrophoresis, and samples showing distinct bands between 400–450 bp were selected for subsequent analysis.

After high-throughput sequencing, the resulting paired-end (PE) reads were spliced using FLASH 1.2.11 software and simultaneously subjected to quality control and filtering. For the optimized sequences, non-repetitive sequences were extracted, and single sequences without repetition were removed. High-quality sequences obtained by the Uparse algorithm were clustered into operational taxonomic units (OTUs) at a 97% similarity level, and representative sequences of OTUs were obtained by removing chimeras during the clustering process. The representative sequences of OTUs were analyzed using the RDP classifier Bayesian algorithm. Bacterial 16S rRNA sequences were compared against the SILVA database (http://www.arb-silva.de), and fungal ITS sequences were compared against the UNITE fungal database (http://unite.ut.ee/index.php).

### 2.4 Statistical analysis

Soil microbial α-diversity was calculated using Mothur (http://www.mothur.org/wiki), and rarefaction curves were generated based on the Shannon index using the R language (version 3.3.1). Principal Coordinate Analysis (PCoA) based on Bray-Curtis distance was performed using QIIME (version 2020.2.0) to assess structural differences in microbial communities. Redundancy Analysis (RDA) and Monte Carlo tests were employed to evaluate the influence of environmental factors on microbial community structure. The impact of environmental factors on microbial community diversity was tested using ordination regression analysis. Bar plots of soil microbial communities, intergroup difference tests, and FAPROTAX functional prediction analysis were conducted and visualized using R software. All data analyses were performed on the free Majorbio I-Sanger Cloud Platform (https://www.majorbio.com). The differences in soil physicochemical properties (pH, EC, SOM, TK, TP, ${\text{NH}}_{4}^{+}$-N, ${\text{NO}}_{3}^{-}$-N, BD, and SWC) and soil enzyme activities (catalase, cellulase, saccharase, and urease) among different vegetation types were assessed using one-way analysis of variance (ANOVA) followed by least significant difference (LSD) multiple comparison test (*P* < 0.05). Unless otherwise specified, all between-group differences reported in Results were statistically significant at *P* < 0.05 based on LSD *post-hoc* tests. Data analysis was completed using SPSS software (version 26.0).

## 3 Results

### 3.1 Physicochemical properties and enzyme activities of rhizosphere soil in halophytes

**Figure 2:**
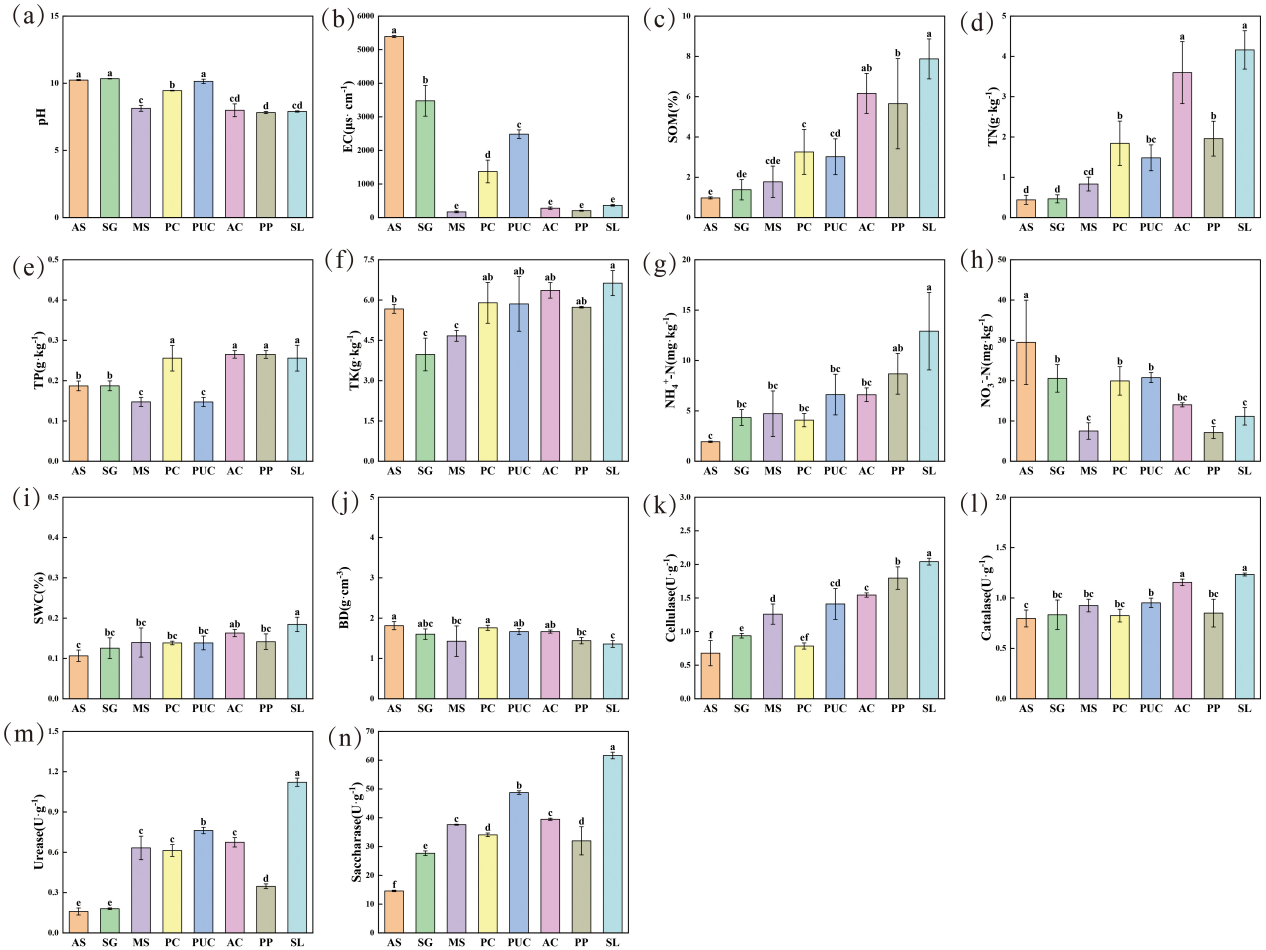
Soil characteristics under different vegetation types. **(a)** pH. **(b)** EC. **(c)** SOM. **(d)** TN. **(e)** TP. **(f)** TK. **(g)** ${\text{NH}}_{4}^{+}$-N. **(h)** ${\text{NO}}_{3}^{-}$-N. **(i)** SWC. **(j)** BD. **(k)** Cellulase. **(l)** Catalase. **(m)** Urease. **(n)** Saccharase. Different letters at the top of each figure indicate significant differences among various halophyte rhizospheres and bulk soil (*P* < 0.05, Duncan test).

The physicochemical properties of rhizosphere soil varied significantly among different vegetation types (Figures 2a–j). All vegetation types significantly increased the contents of SOM, TN, and ${\text{NH}}_{4^{+}}$-N, while reducing ${\text{NO}}_{3^{-}}$-N content and EC. However, the effects of different vegetation types on soil physicochemical properties varied considerably. The SOM levels across vegetation types followed the order: SL > AC > PP > PC > PUC > MS > SG > AS, while TN levels followed: SL > AC > PP > PC > PUC > MS > SG > AS. The pH of all soil samples exceeded 7.8, indicating alkaline soil. Among them, *Populus davidiana* × *P. alba var. Pyramidalis* (PP) had the most significant effect on soil pH, with a pH of 7.82. *Salix linearistipularis* (SL) showed the best improvement in Physical and chemical properties of saline-alkali soils, with high levels of SOM, TN, TP, and TK, and lower EC and pH. Among all vegetation types, SWC and BD showed no significant differences.

Soil enzyme activity is an important indicator of soil health. Compared to Alkaline spot (AS), different vegetation types significantly enhanced soil enzyme activities to varying degrees (Figures 2k–n). For example, sucrase and cellulase activities were significantly increased in all vegetation types. The catalase activities of *Aneurolepidium chinense* (AC) and SL were 1.16 U/g and 1.23 U/g, respectively, significantly higher than AS. Except for *Suaeda glauca* (SG), the urease activities of other vegetation types were significantly increased.

### 3.2 Composition and differences of rhizosphere microbial communities in salt-tolerant plants

**Figure 3:**
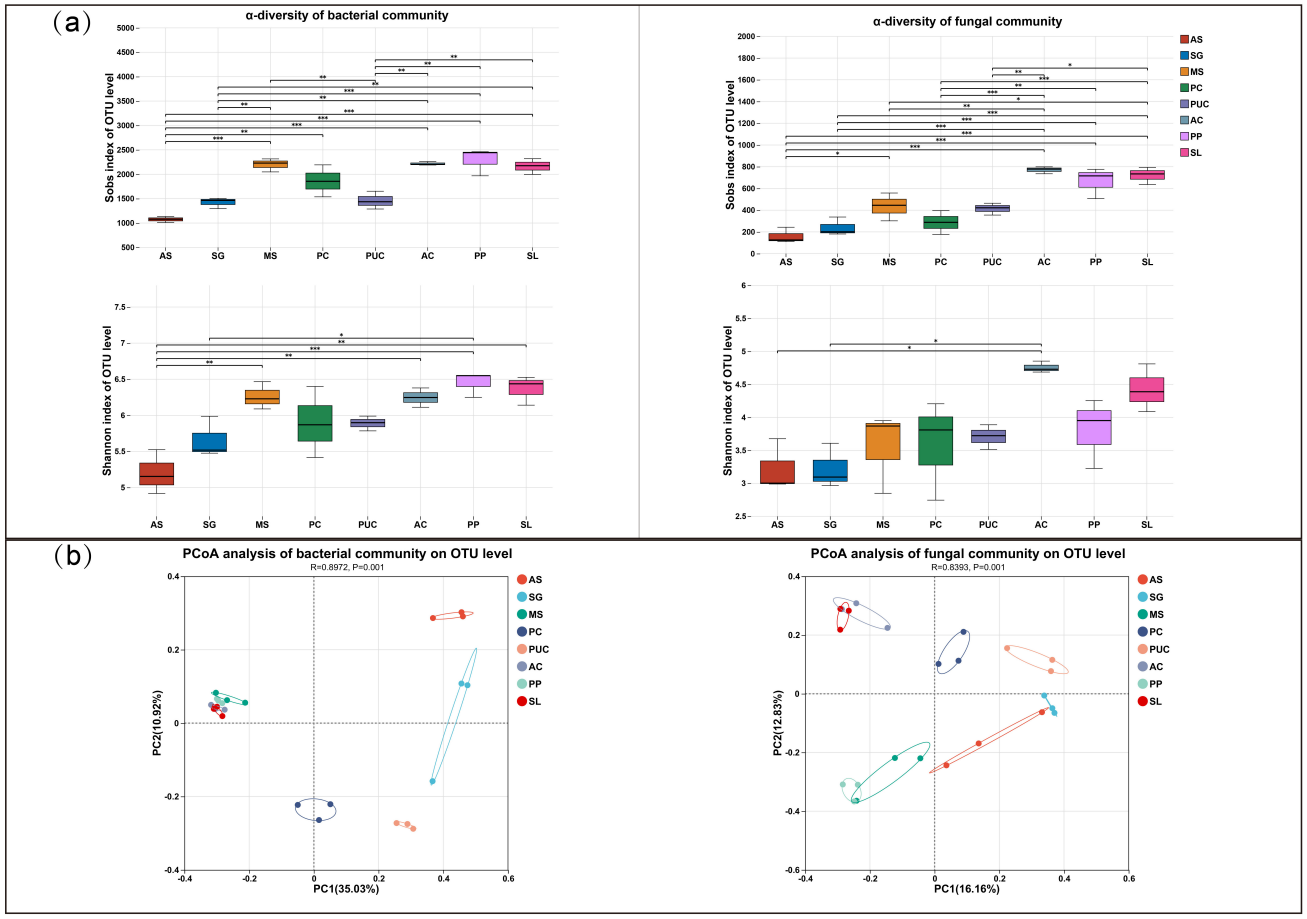
Diversity and differential analysis of rhizosphere microbial communities under different vegetation types. **(a)** *S*_*obs*_ and Shannon index. **(b)** Principal coordinate analysis (PCoA). Significant differences: *p < 0.05, **p < 0.01, ***p < 0.001.

A total of 1,115,143 optimized bacterial sequences and 1,488,479 optimized fungal sequences were obtained from the soil samples. The coverage indices for both fungi and bacteria exceeded 97% (Supplementary Figure 1), indicating that the microbial community sequences obtained were of high quality. The rarefaction curves (Supplementary Figure 2) showed that all samples reached a plateau, suggesting that the sequencing depth was sufficient for subsequent analysis. The diversity and richness of rhizosphere microbial communities in different salt-tolerant plants were evaluated based on the *S*_*obs*_ and Shannon index from α-diversity analysis (Figure 3a). The results demonstrated that: for bacterial communities, both *S*_*obs*_ and Shannon index were significantly higher in the vegetated rhizosphere compared to AS; for fungal communities, while the Shannon index showed significant differences between vegetated and AS areas, no significant variation was observed in *S*_*obs*_. These findings reveal distinct vegetation-type effects on rhizosphere bacterial vs. fungal communities. Principal Coordinates Analysis (PCoA) revealed significant differences in microbial community structure among samples (*p* = 0.001). As shown in Figure 3b, distinct separation was observed between bacterial and fungal communities across different samples. The AS samples exhibited pronounced differentiation from other vegetation types in the PCoA ordination space.

**Figure 4:**
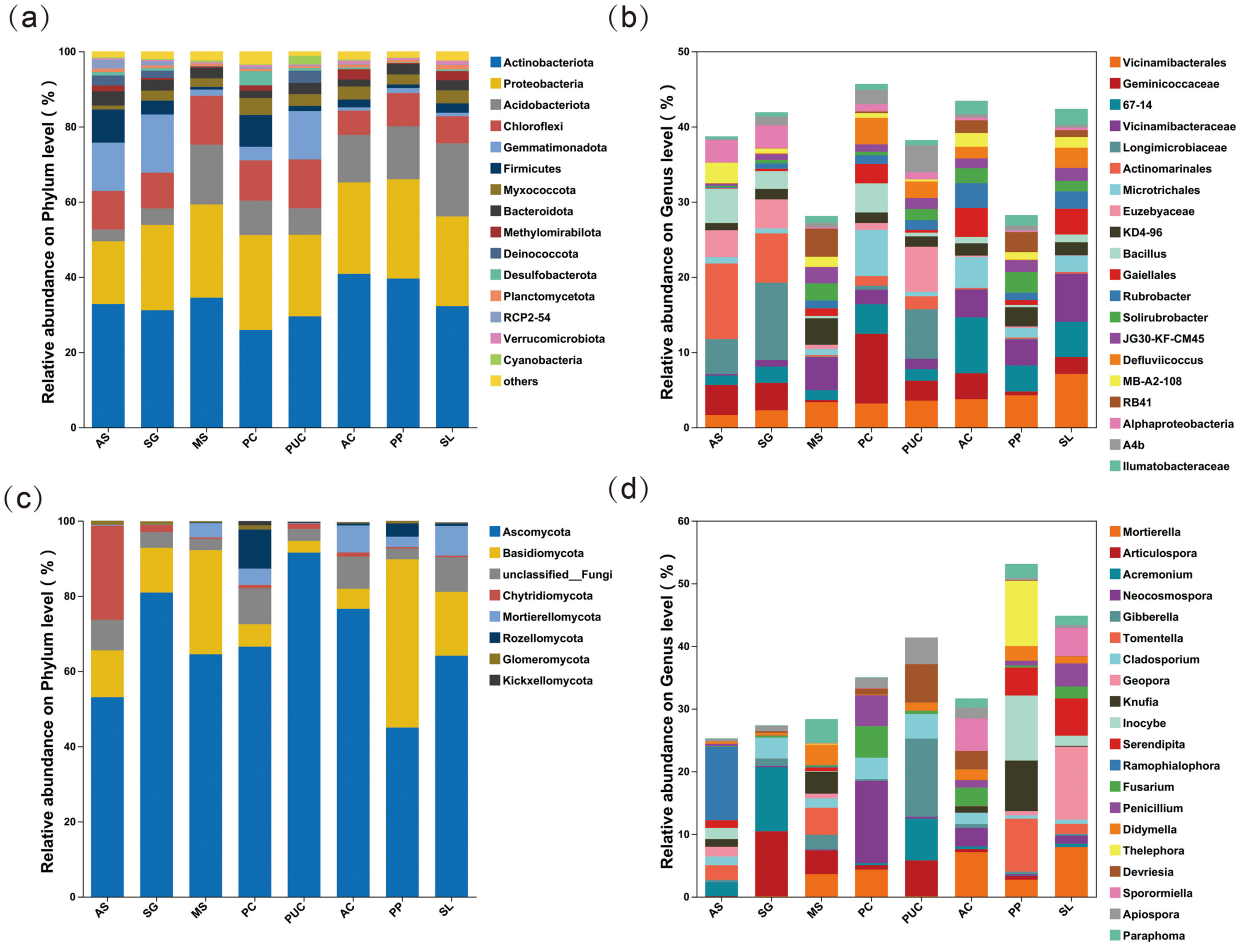
Composition of rhizosphere microbial communities across different vegetation types **(a)** Bacterial community composition at the phylum level. **(b)** Fungal community composition at the phylum level. **(c)** Relative abundance of the top 20 bacterial genera. **(d)** Relative abundance of the top 20 fungal genera.

By analyzing the unique and shared soil microbial OTUs among different samples at the OTU level using Venn diagrams (Supplementary Figure 3), the results showed that AS accounted for 19.37% (436/2251) of the total bacterial OTUs analyzed, while the shared OTUs among salt-tolerant plants represented 9.60% (216/2251). Among these, *Phragmites communis* (PC) had the highest number of unique OTUs. For fungi, 35 OTUs were shared across all samples, and the number of unique OTUs in salt-tolerant plants was consistently higher than that in AS. AS had the fewest unique OTUs, with only 90. The analysis of microbial community structure at the phylum level (Figures 4a, c) revealed the presence of 15 dominant bacterial phyla (relative abundance > 1%) and 8 dominant fungal phyla (relative abundance > 1%) in the rhizosphere soil. Among the 15 dominant bacterial phyla, Actinobacteriota and Proteobacteria emerged as the predominant lineages, collectively comprising 49.50% to 66.01% of the total bacterial community. Compared to AS (Supplementary Figure 4), the relative abundances of Acidobacteriota, and Myxococcota were higher in the rhizosphere of plants, while Firmicutes and RCP2-54 were lower. Among the eight dominant fungal phyla, Ascomycota exhibited the highest relative abundance. The relative abundance of Mortierellomycota increased significantly, whereas that of Chytridiomycota decreased.

Furthermore, the microbial community structure was analyzed at the genus level, with the top 20 genera in relative abundance classified as dominant genera. These top 20 genera accounted for 28.02% to 45.67% of the total bacterial abundance and 25.28% to 53.12% of the total fungal abundance (Figures 4b, d). Among them, the dominant bacterial genera in AS were *Actinomarinales* (10.04%), *Geminicoccaceae* (5.12%), and *Longimicrobiaceae* (4.66%). Notably, *Longimicrobiaceae* was the most dominant bacterial genus in SG and *Puccinellia chinampoensis* (PUC). *Vicinamibacteraceae* exhibited high abundance in *Medicago sativa* (MS), PP, and SL, accounting for 4.38, 3.47, and 6.4%, respectively, while *Geminicoccaceae* (9.27%) *and 67–14* (7.44%) were the most abundant bacterial genera in SG and AC, respectively. For fungi, *Ramophialophora* (11.69%) was the dominant genus in AS, whereas *Articulospora* (10.35%) and *Acremonium* (10.21%) predominated in the rhizosphere of SG. Additionally, *Mortierella* held a significant presence in the rhizosphere fungal communities of MS, PC, AC, PP, and SL.

### 3.3 Interrelationships between soil physicochemical properties and microbial communities

**Figure 5:**
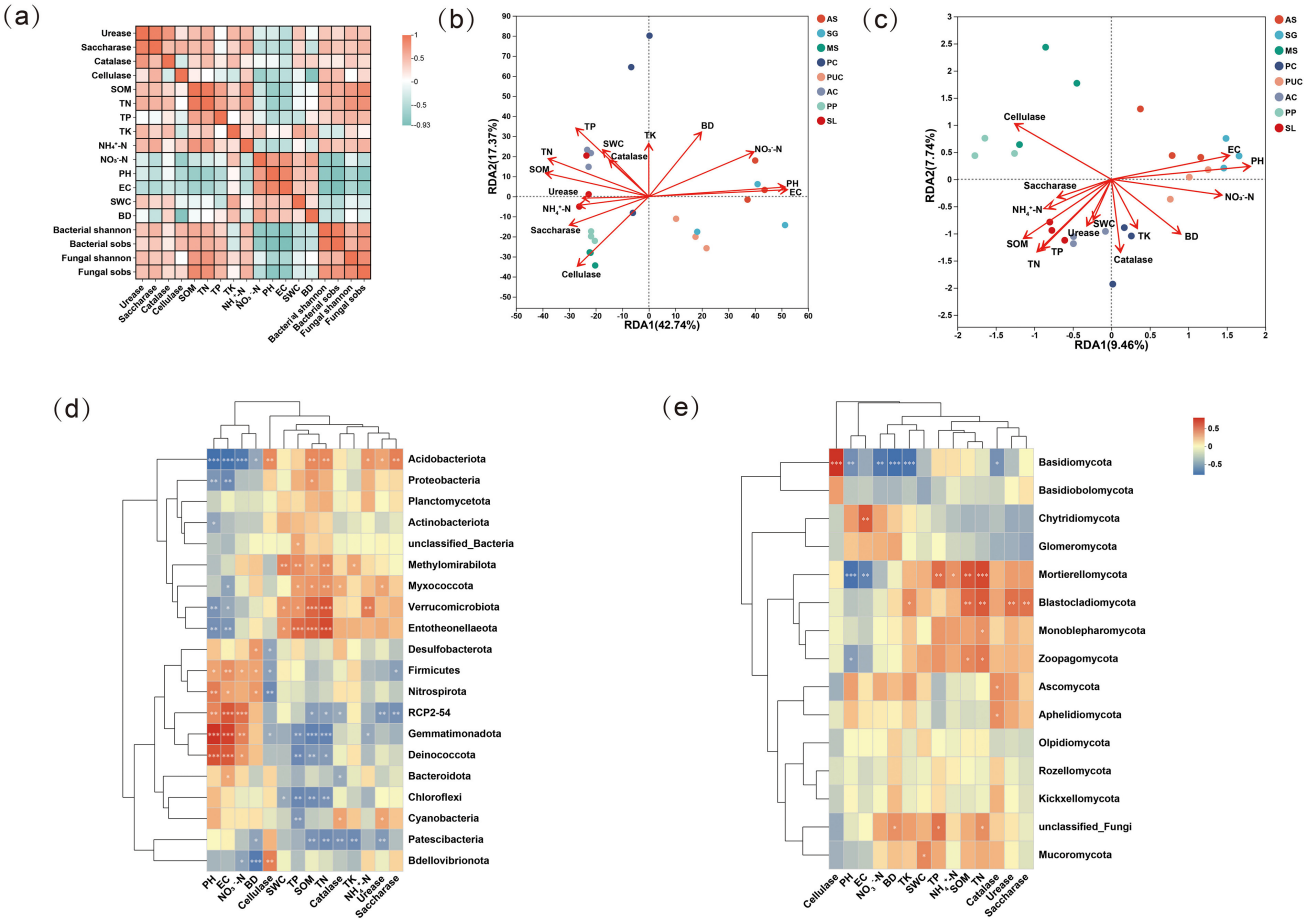
Interaction between environmental factors and microbial communities. **(a)** Pairwise interactions between environmental factors and microbial diversity. **(b)** Redundancy analysis (RDA) for bacteria. **(c)** Redundancy analysis (RDA) for fungi. **(d)** Interaction between the top 20 bacterial phyla and environmental factors. **(e)** Interaction between the top 20 fungal phyla and environmental factors.Significance levels: *p < 0.05, **p < 0.01, ***p < 0.001. The range from red to blue represents the relative abundance from high to low.

Correlation analysis revealed the relationships among soil physicochemical properties, soil enzyme activities, and the richness and diversity of soil microbial communities (Figure 5a). Soil enzyme activities showed a significant negative correlation with soil EC, pH and, ${\text{NO}}_{3^{-}}$-N while exhibiting a positive correlation with fundamental soil physicochemical indicators (TN, TP, TK, and ${\text{NH}}_{4^{+}}$-N). Additionally, the richness and diversity of fungi and bacteria (Shannon index and *S*_*obs*_) were significantly negatively correlated with pH and EC (*p* < 0.05). This indicates that high pH and EC can influence fundamental soil physicochemical properties to some extent and significantly affect the richness and diversity of soil microbial communities.

Redundancy Analysis (RDA) models and Monte Carlo tests (Figures 5b, c, and Supplementary Table 2) were employed to examine the relationships between soil physicochemical properties and soil microbial community composition. For bacterial communities, EC (*R*^2^ = 0.8778; *P* = 0.001) was the most significant factor influencing community structure, followed by pH, ${\text{NO}}_{3^{-}}$-N, and TP. For fungal communities, pH (*R*^2^ = 0.8661; *P* = 0.001) was the primary factor, followed by cellulase activity, TN, and EC. Additionally, pH and EC were positively correlated with salt-tolerant or oligotrophic microorganisms (Figures 5d, e), such as the bacterial phyla Firmicutes, Nitrospirota, RCP2-54, Gemmatimonadota, Deinococcota, Bacteroidota, and Chloroflexi, as well as the fungal phyla Chytridiomycota, Glomeromycota, Ascomycota, and Aphelidiomycota. When soil pH and EC decreased and nutrient levels increased, the abundance of these microorganisms declined. Our results indicate that different microbial communities in saline-alkali soils are sensitive to soil physicochemical properties.

**Figure 6:**
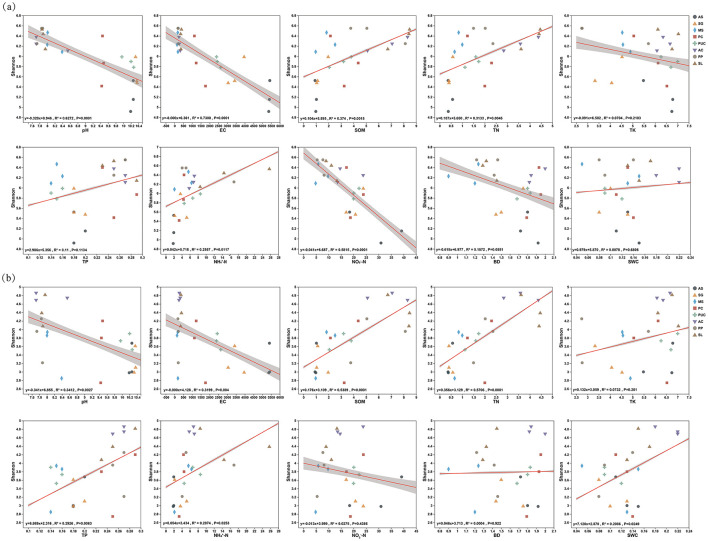
Correlation analysis between shannon index of bacterial and fungal communities and environmental factors. **(a)** Bacterial communities. **(b)** Fungal communities.

Microbial diversity is crucial for ecosystem stability. Using the Shannon index as an indicator, we identified the primary factors influencing soil fungal and bacterial diversity through ordination regression analysis of environmental factors (Figure 6). A decrease in EC was the driving factor affecting bacterial community diversity (*R*^2^ = 0.7308, *P* = 0.0001), while an increase in SOM was a significant factor enhancing bacterial diversity (*R*^2^ = 0.374, *P* = 0.0015). In contrast, TN was the main factor increasing fungal diversity (*R*^2^ = 0.5706, *P* = 0.0001).

### 3.4 Changes in rhizosphere microbial community functions

**Figure 7:**
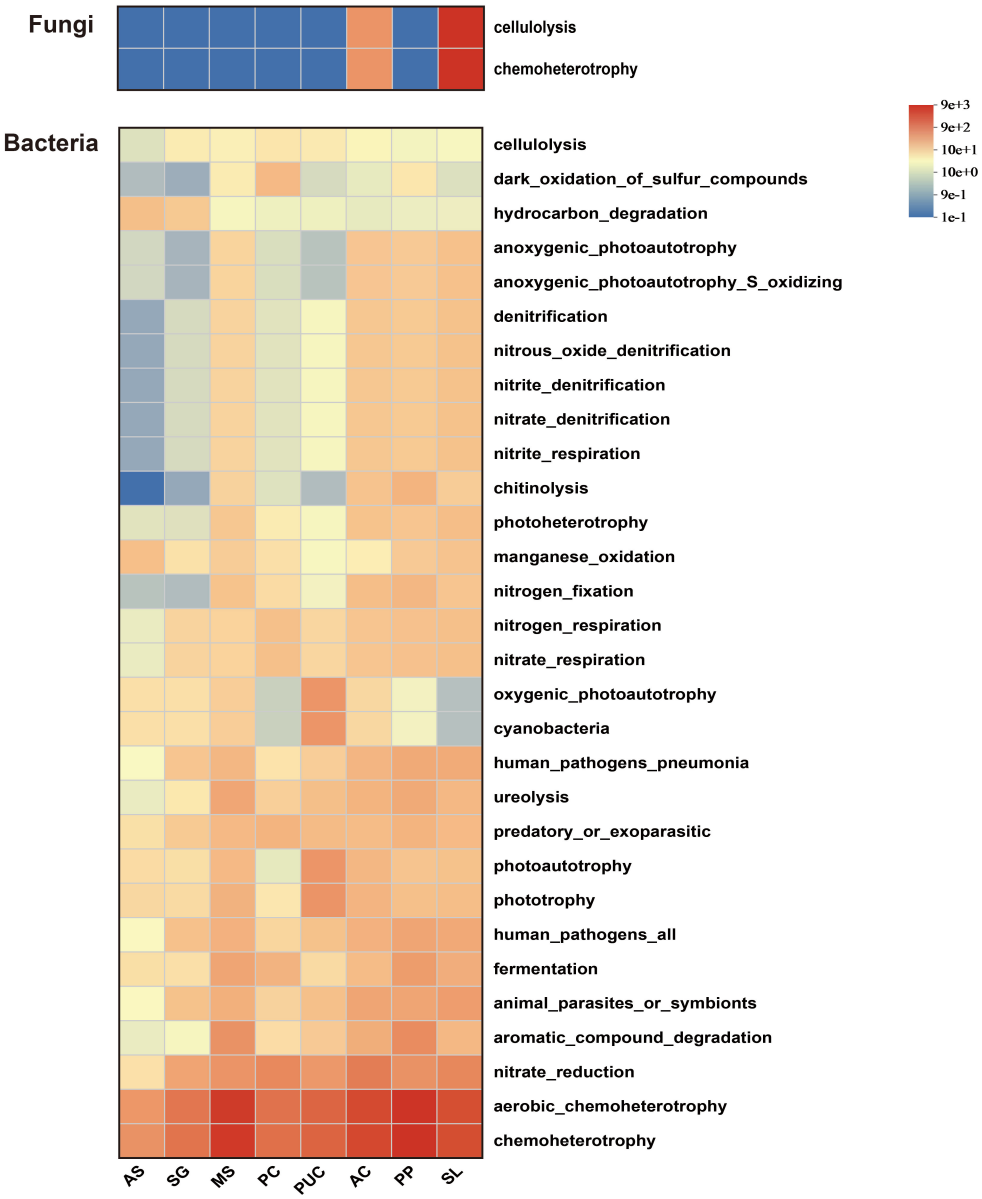
Prediction of the top 30 functional groups in bacterial and fungal communities using FAPROTAX. The range from red to blue represents the relative abundance from high to low.

To further understand the potential functions of rhizosphere microbial communities, we conducted FAPROTAX analysis based on existing databases (Figure 7). A total of 2 fungal functional groups were identified, primarily involved in cellulolysis and chemoheterotrophy processes, with no significant changes observed. Among the 65 bacterial functional groups, the most abundant were those primarily involved in carbon (C) cycling, such as chemoheterotrophy and aerobic chemoheterotrophy, followed by nitrate reduction and aromatic compound degradation. Significant changes were observed in certain functional groups involved in material cycling across different treatments. Functional groups associated with C cycling (cellulolysis and chitinolysis), nitrogen fixation, denitrification (denitrification, nitrous oxide denitrification, and nitrite denitrification), and reduction (nitrite reduction) were significantly enhanced compared to AS. Therefore, we conclude that plants alter the composition of their rhizosphere microbial communities, recruiting relevant functional groups that contribute to improving soil properties.

## 4 Discussion

### 4.1 Effects of salt-tolerant plants on soil physicochemical properties and enzyme activities

Soil salinization severely affects plant growth and leads to reduced crop yields. Phytoremediation is an effective strategy for rehabilitating saline-alkali soils (Jesus et al., 2015; Wang et al., 2020). Plant root exudates can not only improve soil structure and promote the dissolution and leaching of salts (Balyan and Pandey, 2024), but also recruit specific rhizosphere microbial communities to enhance plant tolerance to saline-alkali stress (Lei et al., 2025). Additionally, plant root growth increases soil porosity and promotes downward salt leaching (Gong-Neng et al., 2016), while rhizosphere microorganisms (e.g., PGPR and AMF) enhance salt leaching efficiency by secreting extracellular polymeric substances to stabilize soil aggregates (Peng et al., 2025). Plant-microbe interactions also facilitate biological desalination. Plants can absorb dissolved salts (e.g., Na^+^ and Cl^−^ ions) from soil and translocate these ions to specific tissues or cells (such as old leaves and salt glands), thereby preventing salt accumulation around roots and reducing soil salinity (Flowers and Colmer, 2008; Munns and Tester, 2008; Zhao et al., 2020). Concurrently, rhizosphere microbiota mitigate salt accumulation in the root zone through ion immobilization, volatilization, and by facilitating selective K^+^ uptake in plants (Qin et al., 2016). However, the remediation effects and mechanisms of common salt-tolerant plants in the Songnen Plain remain unclear. This study evaluated the soil quality under seven common salt-tolerant plants and a control (AS) in the same region of the Songnen Plain. We found that plants can alter soil pH and EC. As a key indicator of soil salinity, EC significantly decreased across different treatment groups, indicating reduced soil salinity and improved saline-alkali conditions (Figure 2b). MS, PP, and SL showed the most pronounced reduction in salinity, along with the greatest decrease in soil BD. We hypothesize that this may be due to increased soil porosity resulting from root expansion, further reducing salt levels. These plants may promote salt leaching through developed root expansion (reducing BD) and synergistic effects with rhizosphere microorganisms (e.g., organic acid secretion and Na^+^ immobilization).

Salt-tolerant plants not only directly reduce soil salinity but also indirectly improve soil quality by regulating rhizosphere microbial functions. Plant residues (e.g., litter) and root exudates (including organic acids, sugars, and amino acids) provide microorganisms with abundant carbon sources and energy substrates, significantly enhancing the decomposition and mineralization of soil organic matter (Cheng et al., 2014; Liu et al., 2025). Previous studies have demonstrated that salt-tolerant plants (*Medicago sativa, Agropyron elongatum, Sesbania cannabina*) can significantly increase soil nutrient contents, SOM, TN, and TP (Wang et al., 2024b; Zheng et al., 2023), which is closely associated with the metabolic activities of rhizosphere microorganisms (e.g., nitrogen-fixing and phosphate-solubilizing bacteria; Yue et al., 2023). Functional prediction analysis in this study further confirmed this mechanism, revealing a significant increase in the abundance of functional genes related to carbon and nitrogen cycling in the rhizosphere microbial communities (Figure 7). Moreover, soil enzyme activities (e.g., urease, sucrase, cellulase, and catalase) serve as important indicators for evaluating microbial-mediated carbon and nitrogen cycling (Zuccarini et al., 2023). Our results showed that salt-tolerant plants significantly enhanced soil enzyme activities, with the SL treatment exhibiting the most pronounced effects (Figure 2). This improvement may be attributed to the enrichment of highly efficient degrading microbial communities in the rhizosphere. These microorganisms accelerate organic matter transformation through the secretion of extracellular enzymes, thereby further improving soil fertility (Hu et al., 2024; Zuccarini et al., 2023).

### 4.2 Effects of salt-tolerant plants on soil microbial abundance and diversity

The richness and diversity of soil microorganisms are important indicators of soil health (Wagg et al., 2019). Soil physicochemical properties are considered key factors influencing microbial communities, with microbial diversity and richness showing a significant negative correlation with soil salinity and decreasing as salinity increases (Ma et al., 2016; Qadir et al., 2007). This outcome may be attributed to the elevated osmotic pressure caused by high salt levels, which inhibits the activity of many microorganisms due to water deficiency and can even lead to their death (Wang et al., 2010). Only extremophiles (halophiles and alkaliphiles) can adapt to high-salt conditions through osmotic regulation, thereby surviving and growing. Consequently, high salinity and pH significantly reduce microbial richness and diversity. As demonstrated in this study, AS exhibited the highest pH and total salt content, corresponding to the lowest microbial abundance (Supplementary Figure 3).

Numerous studies have shown that plant cultivation reduces soil salinity, increases soil nutrients, and improves the soil environment, thereby enhancing soil microbial diversity and richness (Cuevas et al., 2019; Wang et al., 2021; Xing et al., 2024; Xu et al., 2023). The decomposition of plant litter inputs a significant amount of organic matter into the soil, serving as a vital energy source for soil microorganisms (Gessner et al., 2010). Additionally, plant root activities promote the formation of soil aggregates, optimize soil pore structure, and significantly enhance soil aeration as roots penetrate deeper soil layers, creating a more favorable microenvironment for microbial growth and metabolic activities (Rabot et al., 2018). Meanwhile, root exudates directly influence the composition of rhizosphere microorganisms. For example, *Suaeda salsa* secretes more compounds under high salt stress, thereby increasing the richness of rhizosphere microorganisms (Liu et al., 2020). These favorable conditions provide an excellent environment for microbial growth and reproduction, promoting increases in microbial richness and diversity. The study found that SL exhibited high diversity indices for both fungi and bacteria (Figure 3), likely due to significant differences in root exudates compared to other vegetation, which influenced fungal growth and reproduction. The results of this study indicate that increases in SOM and TN content are key factors driving significant improvements in soil bacterial and fungal diversity (Supplementary Table 1), further confirming the central role of carbon and nitrogen sources as primary energy and nutrient sources for microbial growth and metabolism (Liang et al., 2017). Based on this finding, in saline-alkali soil vegetation restoration practices, optimizing soil carbon and nitrogen input strategies (e.g., straw return, organic fertilizer application, and biochar amendment; Luo et al., 2011; Zhang et al., 2018) can effectively enhance microbial community richness and diversity, thereby improving ecosystem stability.

### 4.3 Effects of salt-tolerant plants on soil microbial structure

The results demonstrate distinct responses of soil microbial communities to different vegetation types in the Songnen Plain. Multiple factors, including rhizosphere exudates, plant litter, and soil physicochemical properties, collectively influence microbial composition (Buyer et al., 2010). Variations in both quality and quantity of litter and root exudates were observed among different vegetation types. Consequently, although the phylum-level composition of soil microbial communities remained generally consistent across vegetation types, significant differences were observed in the relative abundance of constituent taxa.

In this study, bacterial communities were predominantly composed of Actinobacteriota, Acidobacteriota, Proteobacteriota, Chloroflexi, and Gemmatimonadetes, consistent with previous findings (Buyer et al., 2010; Campbell and Kirchman, 2013). Actinobacteriota emerged as the dominant phylum under stress conditions due to their capacity to degrade recalcitrant organic compounds (Yaradoddi and Kontro, 2021). Notably, *Streptomyces* and *Frankia* within Actinobacteriota contributed to soil health through organic matter decomposition, pathogen antagonism, and nitrogen cycling (Dhanasekaran, 2016; Mitra et al., 2022). Our results indicate that vegetation establishment significantly increased the relative abundance of Actinobacteriota, concomitantly enhancing soil nutrient accumulation. *Proteobacteriota*, comprising predominantly facultative or obligate aerobes, exhibited broad environmental adaptability and consequently maintained high abundance across all soil types (Yang et al., 2014). Several taxa within this phylum (e.g., *Rhizobium, Bradyrhizobium, Azospirillum*) demonstrated nitrogen-fixing capabilities (Kim et al., 2021; Timofeeva et al., 2023). Acidobacteriota played a crucial role in organic matter cycling among plants, fungi, and insects, with their relative abundance showing strong positive correlation with soil organic matter content, aligning with previous reports (Ward Naomi et al., 2009). In our study, pH emerged as a primary determinant of bacterial composition, with Acidobacteriota representing only 3.19% in CK and displaying significant negative correlation with pH values. This observation may reflect the capacity of certain Acidobacteriota members to degrade cellulose with concomitant acetate production (Pankratov et al., 2012). Gemmatimonadetes reached maximal abundance in CK samples with highest salinity, with progressive decline following vegetation establishment, suggesting osmoregulatory adaptation to extreme environments, as reported elsewhere (Gao et al., 2021).

Fungal communities were predominantly composed of Basidiomycota and Ascomycota, which exhibited high relative abundance across all vegetation types. As the dominant functional groups in soil microbial communities, these two phyla play crucial roles in organic matter decomposition and significantly influence global biogeochemical cycles (Zeng et al., 2020). Within Basidiomycota, *Phanerochaete* and *Pleurotus* demonstrated efficient lignin degradation through the secretion of laccase and manganese peroxidase, converting lignin into water and carbon dioxide (Hormiga et al., 2008; Martinez et al., 2001). This enzymatic activity likely contributes to the observed positive correlation between Basidiomycota abundance and cellulase activity (Figure 5). In Ascomycota, *Penicillium* species not only enhanced plant tolerance to abiotic stress (Chaudhary et al., 2018) but also exhibited cellulose-degrading capacity (Rastegari, 2018). Our study revealed significantly higher abundance of *Penicillium* in PC and SL treatments. *Cladosporium*, another prevalent Ascomycete genus, promoted plant growth through indole-3-acetic acid (IAA) production and phosphate solubilization (Răut et al., 2021). Notably, *Mortierella* (representing Mortierellomycota) displayed unique ecological adaptations to saline-alkali stress. Through organic acid secretion and symbiotic associations, *Mortierella* significantly improved host plant phosphorus uptake efficiency (Ozimek and Hanaka, 2021). In contrast, Chytridiomycota, a plant-pathogenic fungal group, showed marked reduction in relative abundance following vegetation establishment (van de Vossenberg et al., 2019).

## 5 Conclusion

The seven salt-tolerant plants studied all demonstrated varying degrees of improvement in soil physicochemical properties, with *Salix linearistipularis* (SL) showing the most significant reduction in soil salinity and enhancement in soil nutrients compared to other vegetation types. Plant cover altered the α-diversity of soil microorganisms in saline-alkali soils, with SL exhibiting high levels of both the Shannon and *S*_*obs*_ indices. EC and TN content were identified as key factors driving differences in bacterial and fungal diversity. The remediation effects of the seven plants led to varying changes in microbial community structure, with EC being the primary factor influencing bacterial community structure. Under different vegetation restoration models, soil pH also decreased to varying degrees, with pH serving as a driving factor for changes in fungal community structure.

In summary, the findings highlight the significant potential of plants in the remediation of saline-alkali soils. Comparative analysis revealed that SL outperformed the other six tested plants in terms of soil improvement and ecosystem stability, making it particularly suitable for the remediation of soda saline-alkali soils.

## Data Availability

The original contributions presented in the study are publicly available. This data can be found at: https://www.ncbi.nlm.nih.gov/, accession numbers PRJNA1267771 and PRJNA1267766.
