# Supplementary material for: The impact of salt-tolerant plants on soil nutrients and microbial communities in soda saline-alkali lands of the Songnen plain
Source: Front Microbiol. 2025 Jun 5;16:1592834. doi: 10.3389/fmicb.2025.1592834 (PMC12177719; doi:10.3389/fmicb.2025.1592834)
Supplement: Supplementary file 1 [file Data_Sheet_1.docx]

Supplementary Material

# Supplementary Tables

Table S1 Monte Carlo Test of the Effects of Soil Physicochemical Properties on Bacterial and Fungal Community Structures in Salt-Tolerant Plants.

|  | Bacteria | | fungi | |
| --- | --- | --- | --- | --- |
|  | R2 | P_value | R2 | P_value |
| EC | 0.8778 | 0.001 | 0.6604 | 0.001 |
| PH | 0.8539 | 0.001 | 0.8661 | 0.001 |
| NO3--N | 0.6411 | 0.001 | 0.5545 | 0.001 |
| TP | 0.5743 | 0.001 | 0.6218 | 0.001 |
| Cellulase | 0.5721 | 0.001 | 0.6687 | 0.001 |
| TN | 0.5612 | 0.001 | 0.6852 | 0.001 |
| SOM | 0.5228 | 0.001 | 0.6333 | 0.002 |
| BD | 0.4137 | 0.006 | 0.4558 | 0.001 |
| Saccharase | 0.3378 | 0.014 | 0.1498 | 0.173 |
| SWC | 0.2472 | 0.05 | 0.1428 | 0.208 |
| NH4+-N | 0.2275 | 0.073 | 0.2448 | 0.043 |
| Urease | 0.1936 | 0.11 | 0.1993 | 0.094 |
| TK | 0.1927 | 0.112 | 0.222 | 0.058 |
| Catalase | 0.1591 | 0.166 | 0.4438 | 0.007 |

## Supplementary Figures


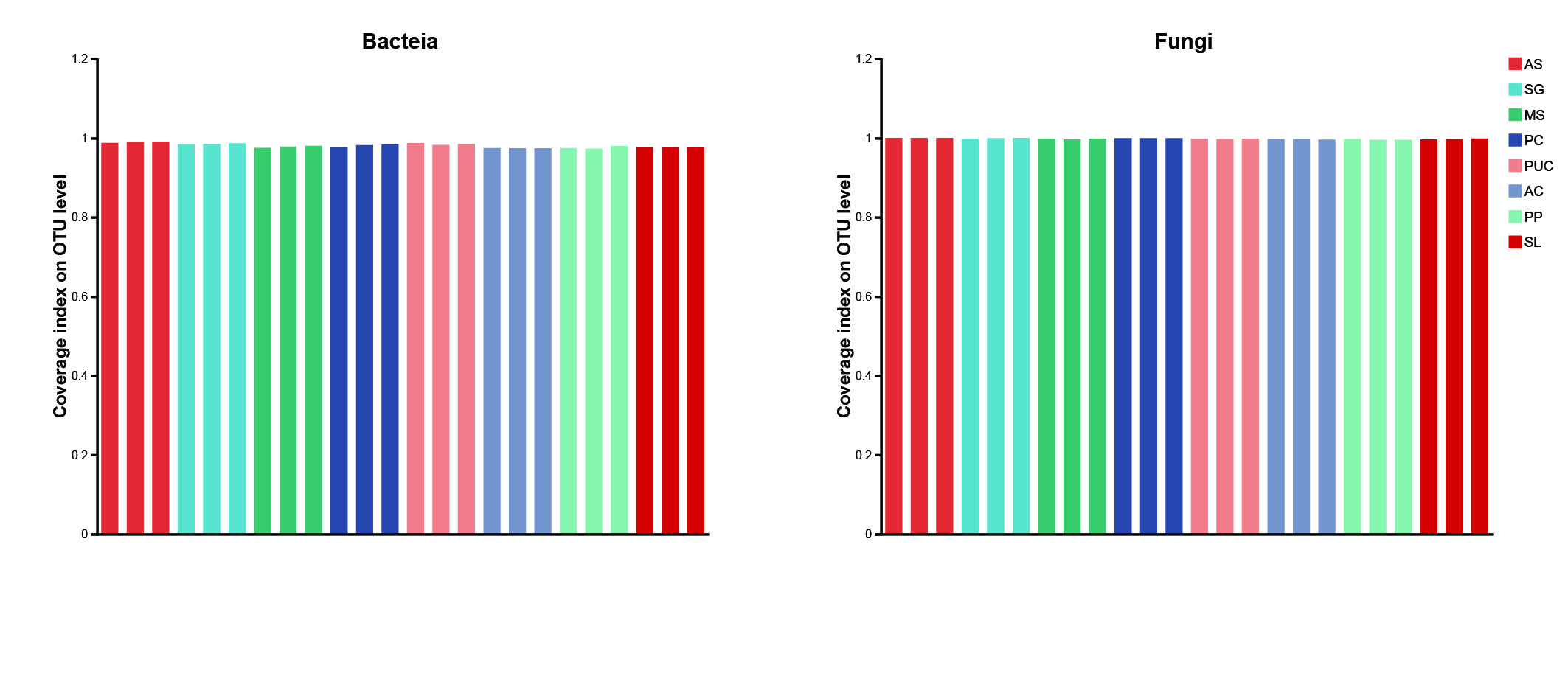


Figure S1 Coverage Index of Rhizosphere Bacteria and Fungi in Salt-Tolerant Plants.


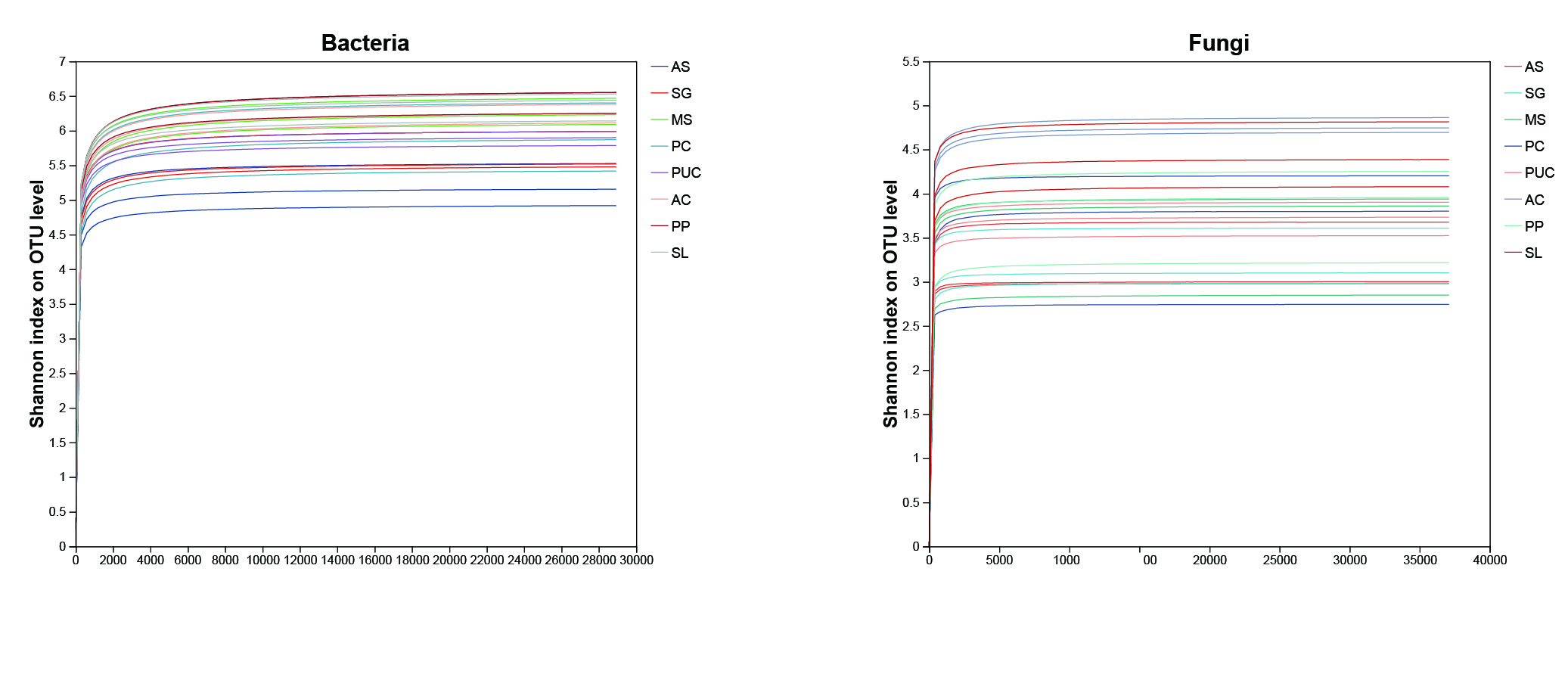


Figure S2 Rarefaction Curves of Bacteria and Fungi in Salt-Tolerant Plants.


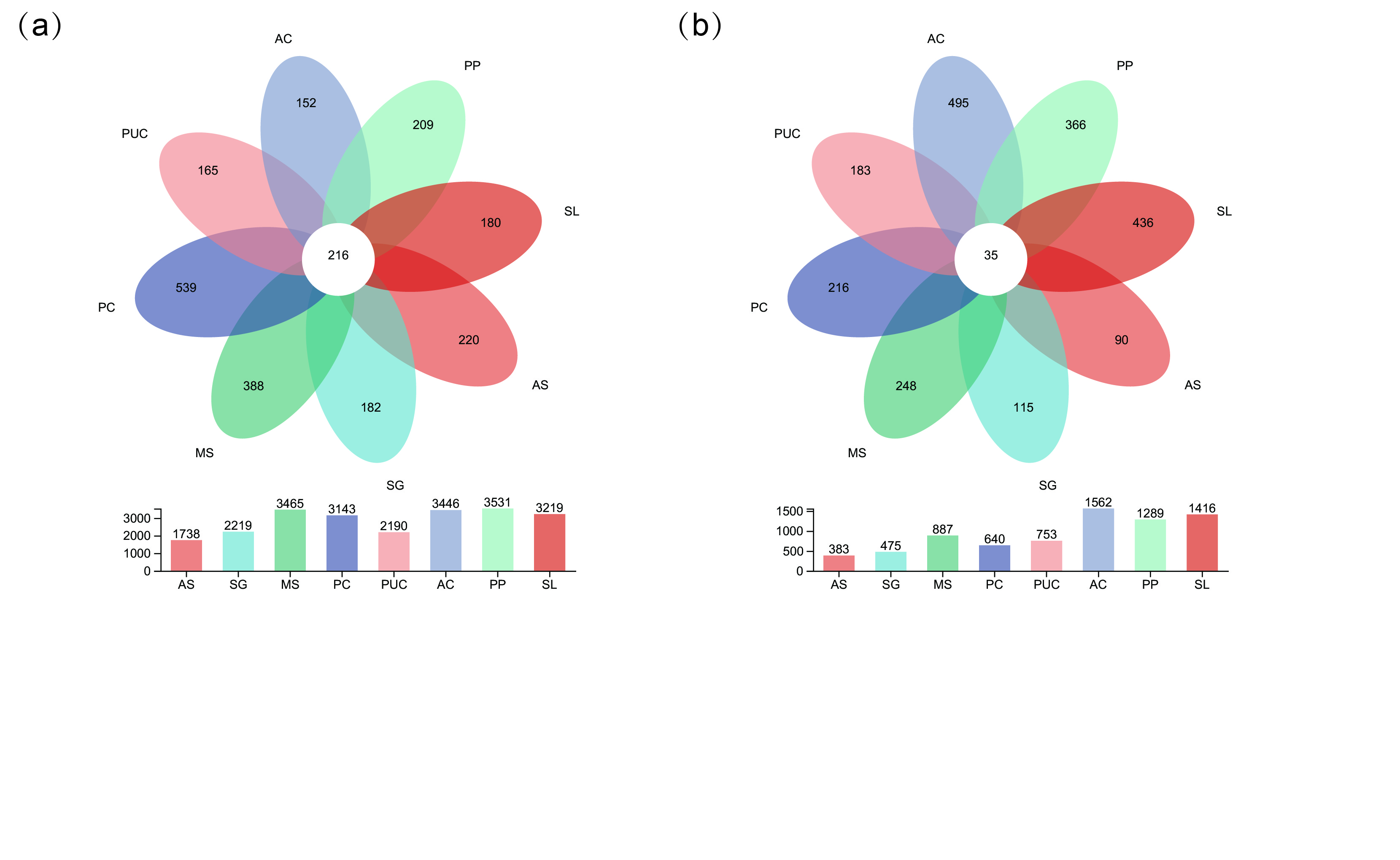


Figure S3 Venn Diagram Analysis of Bacterial (a) and Fungal (b) Species at the OTU Level. Note: The petals represent the number of species unique to each corresponding group, while the center shows the number of species common to all groups. The y-axis of the bar plot indicates the total number of species per group/sample at the selected taxonomic level.


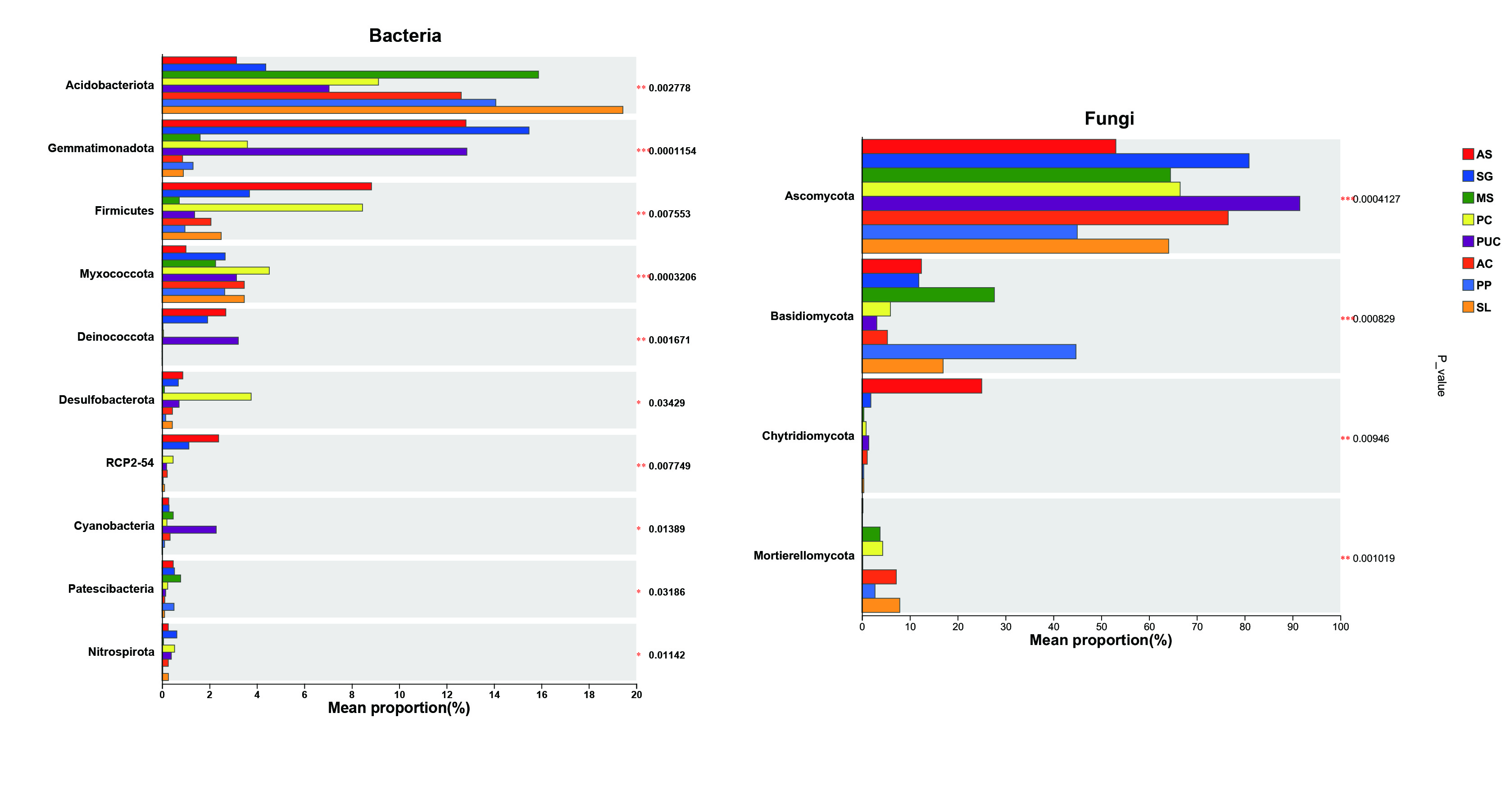


Figure S4 Comparative Analysis of Bacterial and Fungal Community Composition at the Phylum Level.
